# Unveiling Food Avoidance Among Patients With Inflammatory Bowel Disease: Patterns and Pathways to Better Dietary Management

**DOI:** 10.1155/jonm/3669996

**Published:** 2026-02-03

**Authors:** Qingyu Wang, Meijing Zhou, Sha Li, Hana F. Zickgraf, Jiefeng Yang, Yang Lei, Zheng Lin

**Affiliations:** ^1^ Department of Cardiology, Affiliated People’s Hospital of Jiangsu University, Zhenjiang, 212002, Jiangsu, China, ujs.edu.cn; ^2^ Department of Endocrinology, The First Affiliated Hospital With Nanjing Medical University, Nanjing, 210029, Jiangsu, China, njmu.edu.cn; ^3^ School of Nursing, Nanjing Medical University, Nanjing, 211166, Jiangsu, China, njmu.edu.cn; ^4^ Department of Research, Rogers Behavioral Health, Research Center, Oconomowoc, 53066, Wisconsin, USA; ^5^ Department of Gastroenterology, The First Affiliated Hospital With Nanjing Medical University, Nanjing, 210029, Jiangsu, China, njmu.edu.cn; ^6^ Department of Nursing Administration, The First Affiliated Hospital With Nanjing Medical University, Nanjing, 210029, Jiangsu, China, njmu.edu.cn

**Keywords:** food avoidance, inflammatory bowel diseases, latent profile analysis, nutrition, root cause analysis

## Abstract

**Aim:**

To investigate potential types of food avoidance among patients with inflammatory bowel disease (IBD) and identify the contributing factors.

**Background:**

Food avoidance may be an important risk factor for poor physical and mental health in patients with IBD. However, there is limited research on food avoidance within the Chinese context.

**Methods:**

Between July 2022 and December 2023, patients with IBD during appointment at the First Affiliated Hospital with Nanjing Medical University was investigated with paper questionnaires to assess food avoidance, food category avoidance, fear of disease progression, negative illness perception, IBD‐related self‐efficacy, and social support. Demographic and disease‐related characteristics were also collected. Latent profile analysis (LPA) was used to examine food avoidance in patients with IBD, and the correlates were investigated using regression analysis.

**Results:**

LPA showed that respondents could be classified into three groups in terms of food avoidance, namely, the mild‐food avoidance adaptation group (*n* = 72, 22.29%), the moderate‐food pleasure deficiency group (*n* = 163, 50.46%), and the severe‐food avoidance impairment group (*n* = 88, 27.24%). The total number of avoided foods in the three groups was 2.78 ± 2.37, 3.59 ± 2.49, and 3.89 ± 2.51, respectively (*p* = 0.02). In the multinomial logistic regression analysis, higher levels of fear of disease progression and negative perceptions were associated with the severe avoidance group. In contrast, patients in remission were more likely to fall into the mild and moderate avoidance groups.

**Conclusion:**

Patients with IBD may exhibit long‐term, spontaneous food avoidance, which often presents at high levels. Furthermore, patients with IBD exhibit considerable heterogeneity in their food avoidance patterns, categorizing them into three distinct categories. Future dietary management strategies should be tailored based on the specific characteristics and predictive factors of these food avoidance patterns.

**Implications for Nursing Management:**

Given the prevalence and heterogeneity of food avoidance in patients with IBD, nurse managers should implement stratified interventions tailored to patient characteristics. Training nurses in culturally sensitive dietary education and emotional regulation strategies may improve the management of food‐related behaviors and support patients’ adaptive coping with the disease.

## 1. Introduction

\Inflammatory bowel disease (IBD) is a category of chronic, intestinal conditions, including ulcerative colitis (UC) and Crohn’s disease (CD), which frequently manifests with intestinal symptoms such as diarrhea, stomach pain, mucopurulent and bloody feces, and severe abdominal discomfort [1]. The number of global IBD cases increased from approximately 3.32 million in 1990 to about 4.90 million in 2019, a significant rise of 47.45% [2]. Due to its unknown cause, recurrent episodes, and the fact that there is currently no cure, patients with IBD require lifelong management of the disease from the time of diagnosis [1].

Dietary management is the preferred disease management goal for patients with IBD, who often choose to avoid foods that are perceived as triggering symptoms to cope with the disease [3]. Food avoidance refers to the practice of deliberately avoiding certain foods or food categories [4]. Patients with IBD frequently avoid certain food categories, averaging 6.9 categories [5]. At least 86% and 74% of patients avoid at least one food category during the acute and remission phases, respectively [6, 7]. Studies reveal that patients with IBD frequently avoid specific foods or food categories, usually because of their own beliefs rather than true dietary allergies or intolerances [8, 9]. A systematic review found that 28%–89% avoid certain foods and 41%–93% eat restrictively [6]. Second, because of the difficulty of obtaining professional guidance, patients often avoid food based on self‐experimentation and continue to expand the types of food avoidance as symptoms recur [10]. Food avoidance in patients with IBD is accompanied by constant adjustment of dietary choices, reducing the variety of food intake, avoiding once‐favorite foods, and eating foods that are different from those of family and friends [11–13]. Such negative experiences can lead to frustration, anxiety, depression, malnutrition, and social alienation, among other adverse consequences [11–13]. In recent years, the academic focusing on food avoidance among patients with IBD has primarily centered on identifying specific avoidance foods and their negative impacts. Therefore, this study focuses on exploring the severity of food avoidance in patients with IBD and systematically examining the factors influencing food avoidance.

Research pertaining to food avoidance among patients with IBD has predominantly concentrated on identifying the specific foods avoided. This focus neglects the critical aspects of the severity of food avoidance and the consequent psychosocial detriment. To determine the types of food that patients avoid, traditional investigations have used a food frequency questionnaire [14]. Yet, these instruments, aimed at gauging recent dietary intake, are often unwieldy and prone to recall bias. Moreover, such questionnaires are limited to categorizing the numbers of avoided foods and fail to capture the extent of avoidance and its underlying psychological dimensions. The Fear of Food Questionnaire (FFQ) was specifically designed to assess the intensity and psychosocial impact of food avoidance [15]. Its notable reliability and user‐friendly nature render it a more apt tool for this investigation, particularly in evaluating the severity of food avoidance among patients with IBD [15]. Therefore, this study used the FFQ questionnaire to explore food avoidance in patients with IBD. As previous studies have mostly used food frequency questionnaires to investigate food avoidance, the present study also investigated the number and specific types of foods avoided using this methodology.

Previous studies, most using food frequency approaches to quantify avoidance, have found significant heterogeneity in food avoidance among patients with IBD [16]. However, the specifics of these group differences and the underlying reasons have yet to be fully explored. Latent profile analysis (LPA) presents a more individual‐centric approach [17]. This approach involves generating a categorical variable (profile assignment) that reflects the relationship between continuous variables used to generate the groups [18]. Using common characteristics, it divides people into various groups and maintains the distinction between them [17]. The use of LPA to investigate latent profiles of overall food avoidance and impact has important implications for clinical practice among patients with IBD. Healthcare providers, by analyzing these latent profiles, can more precisely identify patients exhibiting combinations of food avoidance behaviors/impacts that may be associated with specific problematic outcomes. Consequently, this facilitates the customization of treatment strategies to cater to specific subgroups within the IBD patient population.

**FIGURE 1:**
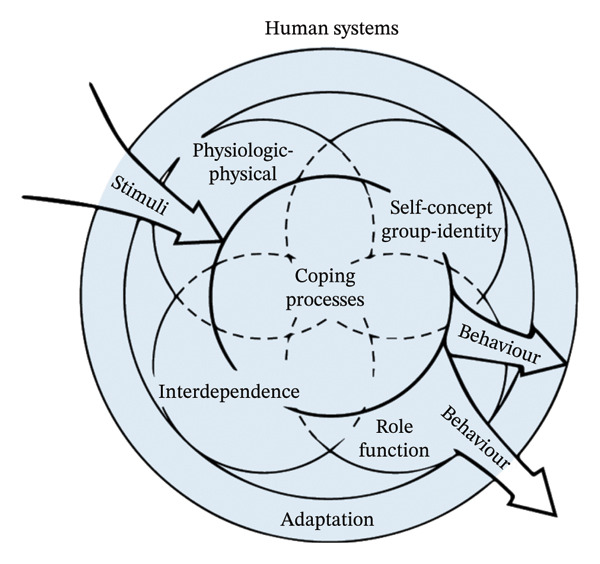
Conceptual framework of the Roy adaptation model.

**FIGURE 2:**
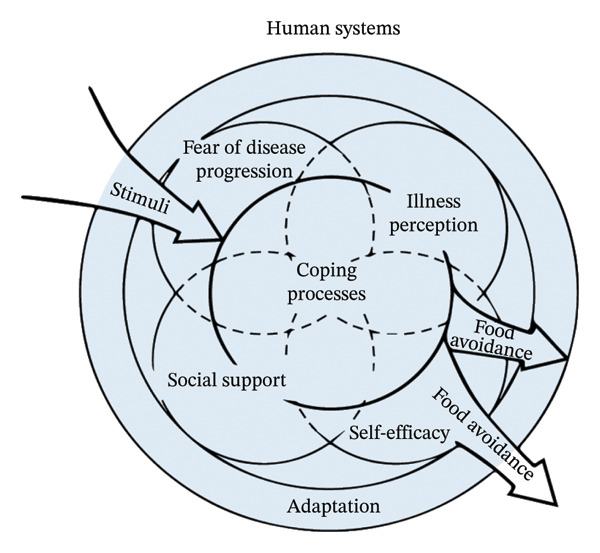
Hypothesized relationships of food avoidance in patients with inflammatory bowel disease based on the Roy adaptation model.

Current quantitative studies related to food avoidance in patients with IBD are fragmented and lack a systematic framework to guide them. Whereas theory‐based research helps to explain potential explanatory mechanisms, the present study systematically explores the influences of food avoidance based on the Roy adaptation model [19]. The Roy adaptation model views the human being as a holistic adaptive system and focuses on the interactions between the person, the environment, and health [20]. It is widely used in health domain research because it helps understand complex and dynamic health issues [21]. The Roy adaptation model considers the adaptive system to include input stimuli, coping mechanisms, adaptive styles, and output effective or ineffective responses [22], as illustrated in Figure 1. Of these, specific expressions of adaptive styles include physiological needs, self‐concept, role function, and interdependence [22]. Avoidance is a key feature of maladaptation [23] (Krypotos et al., 2015). In the face of disease challenges, food avoidance that is self‐initiated, not time‐limited, and negatively reinforced by short‐term reductions in anxiety or distress is conceptualized as a manifestation of maladaptation in patients with IBD. Based on the Roy adaptation model, this study hypothesized that patients with IBD are stimulated by illness, which prompts them to develop illness coping behaviors and to adapt and regulate changes brought about by the illness in terms of physiological needs, self‐concept, role functioning, and interdependence, which ultimately results in food avoidance. Therefore, in this study, disease characteristics, fear of disease progression, illness perception, self‐efficacy, and social support were selected to analyze the influencing factors of dietary avoidance in patients with IBD, and the hypothesized relationships in this study are shown in Figure 2.

Therefore, this study used scientific measurement tools and methods to analyze the different categories of food avoidance in patients with IBD, to describe the food choices of patients with different categories of food avoidance, and to systematically explore the factors affecting the categories of food avoidance according to the Roy adaptation model.

## 2. Methods

### 2.1. Participants

This was a cross‐sectional study and was approved by the Institutional Review Board (IRB) and Ethics Committee of Nanjing Medical University in Jiangsu, China (protocol number: 2022955). Offline paper questionnaires were used at the First Affiliated Hospital of Nanjing Medical University from July 2022 to December 2023.

The inclusion criteria were (1) diagnosis of IBD according to the Chinese Society of Gastroenterology criteria released in 2018 [1]; (2) age of 18 years or older; (3) conscious and no communication disorders; and (4) disease duration of at least three months (this criterion was used because the food frequency questionnaire records food intake over the past three months). The exclusion criteria were (1) combined with malignant tumors or severe organic diseases; (2) diagnosed with cognitive impairment or psychiatric disorders; (3) received enteral nutrition (including gastric or jejunal tubes) support within the past 3 months; (4) pregnant, breastfeeding, or having special dietary requirements; and (4) avoided certain types or categories of food due to religious beliefs. This study adopted the method of multinomial analysis, and 23 variables were considered in this study. This sample size was adequate to power multinomial analyses with up to 23 variables (i.e., minimum *N* = 23∗10) and group comparisons on the FFQ ($n={\left(\mu_{\alpha /2}\sigma /\delta \right)}^{2}$), accounting for an anticipated 20% invalid or missing data. The sample size is at least 288 cases.

### 2.2. Data Collection and Quality Control

To ensure the quality of each phase of the study, specific control measures were implemented: (1) Study design phase: The first author created the survey packet and medical record review procedure after reviewing relevant literature and discussing it with the research team. The final questionnaire was also determined after the presurvey; for questionnaire collection, two researchers were responsible for the collection of the questionnaires after receiving a uniform training and distributing the paper questionnaires face‐to‐face to the target inpatients. (2) Pilot study phase: About 30 questionnaires were anticipated. The study team discussed completion time, status, patient feedback, and analytic results regularly. The study design and issues have been reviewed and modified by relevant specialists. (3) Formal survey phase: About 300 questionnaires were anticipated. Before the survey, the researchers explained the purpose, significance, and requirements of the study to the patients and promised to keep the data confidential. After obtaining informed consent from the patients, a self‐administered questionnaire was distributed; during the survey, the researcher manually viewed the electronic medical record system and filled in the information related to the characteristics of the disease with the help of healthcare professionals. Remaining questions were distributed by the researcher on the spot, and patients filled in the remaining questions on the spot. During the filling process, the researcher answered the questions in a timely manner. All participating patients were given an electronic copy of the IBD care manual at the end; after the survey, if any questions were left unanswered on the questionnaire, researchers verified and supplemented the missing information with the patients on the spot. Questionnaires were recognized as invalid and excluded if there was a pattern of answers and the answers were the same for all questions.

### 2.3. Measures

#### 2.3.1. Patient Characteristics

Demographic characteristics data covered gender, age, educational level, marital status, living in an urban area or not, living alone or not, employment status, monthly household income, and self‐perceived financial stress (categorized as no stress: no burden; moderate stress: manageable burden; and high stress: beyond financial capacity). Disease characteristics data included type of disease, current disease status (using the Simple Clinical Colitis Activity Index and the Harvey–Bradshaw Index), years of diagnosis, bowel surgery status, use of biologic therapy, nutritional support therapy, malnutrition risk (using the Nutritional Risk Screening 2002 assessment), and food tolerance test.

After admission, registered nurses assessed nutritional risk using the Nutritional Risk Screening 2002 score after admission by the registered nurse, and the cut‐score was ≥ 3, indicating that there was a risk of malnutrition in patients [24]. Attending physicians evaluated disease activity. The UC activity was assessed using the Simple Clinical Colitis Activity Index, with scores < 3 indicating remission and ≥ 3 indicating active disease [25]. The CD activity was measured using the Harvey–Bradshaw Index, with scores < 5 indicating remission and ≥ 5 indicating active disease [26]. These data were abstracted from the electronic medical record after patients consented and responded to the paper surveys.

#### 2.3.2. Food Avoidance Intensity and Impact: FFQ

The FFQ was developed in 2022 and was translated into Chinese in 2023 [15, 27]. This scale contains 18 items and assesses five illness‐related coping modes: gastrointestinal symptoms fears (8 items), food avoidance (4 items), food fears (3 items), social impairment (3 items), and loss of pleasure (4 items). The score was answered on a six‐point Likert scale from 0 (strongly disagree) to 5 (strongly agree), and the total score range was 0 to 90, with higher total scores indicating higher food avoidance [15]. Qualitative score ranges are 0–15 (minimal), 16–30 (mild), 31–45 (moderate), and 46–90 (severe) [28]. The Cronbach’s *α* coefficient of this scale was 0.86 in our study.

#### 2.3.3. Food Category Avoidance: The Simple Food‐Frequency Questionnaire (SFFQ)

The SFFQ was simplified and modified from the food‐frequency questionnaire used in the China Nutrition and Health Survey [29]. The original scale incorporated a total of 25 food categories and had good retest reliability and dietary pattern analysis validity. According to dietary guideline recommendations for patients with IBD, sweets, fried foods, processed meat products, beverages, and alcohol should be excluded from the survey of food avoidance categories [30, 31], because these have been shown in studies to potentially exacerbate symptoms or inflammation in patients with IBD [30, 32, 33]. Additionally, healthcare personnel document the patient’s food allergies or intolerances in the electronic medical record upon admission. Researchers subsequently review these records and verify the information with the patients. Foods avoided due to intolerance or allergies were excluded from the survey. The SFFQ was used to collect information on 18 food categories consumed by the patients in the last three months: rice, porridge or rice soup, flour‐based foods (e.g., steamed buns, bread, and noodles), stuffed foods (e.g., buns, wontons, and dumplings), coarse grains (including brown rice, millet, corn, oats, and ormosia), root vegetables (e.g., sweet potatoes, potatoes, and yams), dairy products (e.g., milk, yogurt, and powdered milk), eggs (e.g., chicken eggs, and duck eggs), red meat (e.g., pork, beef, and lamb), poultry (e.g., chicken, duck, and goose), freshwater aquatic animals (e.g., softshell turtle, grass carp, and river shrimp), seafood (e.g., sea cucumber, abalone, and scallops), bean products (e.g., tofu, dried tofu, and vegetarian chicken), nut foods (e.g., walnuts, peanuts, and pistachios), dark‐colored vegetables(e.g., spinach, tomatoes, and carrots), light‐colored vegetables (e.g., cabbage, radish, and cucumber), edible fungi (e.g., button mushrooms, shiitake, and straw mushrooms), and fruits (e.g., apples, pears, and peaches). This questionnaire is widely used in dietary studies of patients with chronic diseases. The total SFFQ score, which ranges from 0 to 18, had a Cronbach’s *α* of 0.69.

#### 2.3.4. Physiologic Needs: Fear of Progression Questionnaire Short Form (FoP‐Q‐SF)

The FoP‐Q‐SF was developed in 2006 and was translated into Chinese in 2015 [34, 35]. It is widely used to examine fear of disease progression and its impact on psychosocial functioning among patients with IBD. This scale consists of 2 dimensions and contains 12 items, including the physiological health dimension (6 items) and the social family dimension (6 items). The items are scored from 1 (never) to 5 (often). The scale is self‐rated by patients with a total score of 12 to 60 points. The higher the score, the higher the patient’s fear of disease progression, and the total score ≥ 34 indicated dysfunctional fear of recurrence. The Cronbach’s *α* coefficient of this scale was 0.92 in our study.

#### 2.3.5. Self‐Concept: Brief Illness Perception Questionnaire (IPQ‐B)

The IPQ‐B was developed in 2006 and was translated into Chinese in 2015 [36, 37]. The IPQ‐B includes 9 items grouped into three dimensions: cognitive illness representations (5 items: consequences, timeline, personal control, treatment control, and identity), emotional representations (2 items: concern and emotional response), and illness comprehensibility (1 item: coherence). Item 9 is an open‐ended question asking respondents to list the three most important factors they believe caused their illness. In this study, only the first eight items were analyzed quantitatively to assess illness perceptions. The ninth item was excluded from analysis due to its qualitative nature. The first eight items were rated on a scale from 0 (minimum) to 10 (maximum). The scale is self‐rated by patients with a total score of 0 to 80 points. The higher the score, the stronger the negative perception of disease. The Cronbach’s *α* coefficient of this scale was 0.60 in our study.

#### 2.3.6. Role Function: IBD‐Self‐Efficacy Scale (IBD‐SES)

The IBD‐SES was developed to measure the self‐efficacy of patients with IBD and was translated into Chinese in 2014 [38, 39]. The Chinese version of the scale contains 29 items. It consists of 4 dimensions, including managing stress and emotions (9 items), managing medical care (8 items), managing symptoms and disease (7 items), and maintaining remission (5 items). The Likert 1 to 10 score method is adopted. The scale is scored from 1 (strongly unconfident) to 10 (very confident), with a possible score of 29∼290. The higher the total score, the stronger the sense of self‐efficacy. The Cronbach’s *α* coefficient of this scale was 0.949 in our study.

#### 2.3.7. Interdependence: Social Support Rating Scale (SSRS)

The SSRS was designed in 1986 and widely used in China [40]. It consists of 10 items, including three dimensions of subjective support, objective support, and availability of support. The scale is graded from 1 to 4. The higher the score, the higher the level of social support. The scale is self‐rated by patients with a total score of 12 to 83 points. The Cronbach’s *α* coefficient of this scale was 0.71 in this study.

### 2.4. Data Analysis

Double data extraction and entry were performed to ensure accuracy. The data were analyzed by IBM SPSS Statistics version 26.0 (IBM Corp., Armonk, NY, USA) and Mplus version 8.3 (Muthén and Muthén, Los Angeles, CA, USA).

First, FFQ items were used for LPA. Model fit was evaluated based on the following fit indices: Akaike information criterion (AIC), Bayesian information criterion (BIC), and adjusted BIC (aBIC). Lower values of AIC, BIC, and aBIC indicate better model fit (Wang, 2014). The Lo–Mendell–Rubin likelihood ratio test (LMR) and the bootstrap likelihood ratio test (BLRT) were examined to evaluate the absolute fit between a *k* − 1 class model and a k‐class model. In the case of a significant *p* value, the *k* potential model was supported, whereas in the absence of a significant *p* value, the *k* − 1 latent profiles were supported. For model classification precision, entropy values greater than 0.80 were considered adequate (Wang, 2014). Finding the best food avoidance model for studying patients with IBD depends on how well the statistical evidence fits with how the model is interpreted in real life. Characteristic attributes of the latent class model were named based on mean line chart fluctuations for each item.

Second, SPSS 26.0 was used to perform descriptive statistics, assess common method bias, and conduct univariate, multicollinearity, and regression analyses. This study’s respondents completed self‐report questionnaires, possibly introducing method bias [41]. Common method bias was assessed using Harman’s single‐factor test [41]. Means and standard deviations (SD) were reported to describe the continuous variables, while frequencies (*n*) and percentages (%) were used to describe categorical variables. The latent classes of food avoidance in patients with IBD were considered the dependent variables. Univariate analysis was used to compare multiple groups. Univariate analysis methods include chi‐square (*χ* ^2^) tests for categorical variables and Fisher’s exact probability for continuous variables. When multiple comparisons were involved, Bonferroni correction was applied to adjust *p* values and control for Type I error [42]. Multicollinearity analysis was performed on statistically significant variables in the univariate analysis. Multicollinearity was high if the variance inflation factor (VIF) was five or more [43]. Variables with high multicollinearity were excluded before performing multinomial logistic regression. Multinomial logistic regression analyses were used to determine the influencing factors of food avoidance in patients with IBD. The independent variables selected for inclusion in the multinomial regression models were those associated with *p* values less than 0.05 in the univariate analysis. Statistical significance was set at *p* < 0.05.

## 3. Results

### 3.1. Information Collection

A total of 330 questionnaires were collected, including 30 from the presurvey. Seven questionnaires were deemed invalid due to apparent response patterns, resulting in 323 valid responses (effective response rate: 97.88%). Based on expert recommendations during the presurvey phase, the collection of specific food category avoidance data was initiated from the 22nd participant. As a result, 302 of the 323 valid questionnaires included information on specific avoided food categories. All 323 valid questionnaires were included in the overall analysis of food avoidance and its related factors.

### 3.2. Assessment of Common Method Bias

Based on the Harman single‐factor test, 21 factors had eigenvalues greater than 1, and the variance explained by the first factor was 21.19%, which was no more than 40% [44]. Therefore, this research showed no serious common method bias problem.

### 3.3. Classification of Latent Profiles

Latent class models were fitted based on the food avoidance assessment data of patients with IBD. In this study, the number of categories was gradually increased from the initial model, and 1–5 potential category models were established sequentially, as shown in Table 1. As the number of categories increased, the AIC, BIC, and aBIC values decreased. The entropy of model 3 was 0.889 > 0.800, and the *p* of LMR and BLRT were < 0.05. In model 3, the number of patient cases in each category was 72, 163, and 88, respectively, and the number of cases in each category was > 50. Considering the practical significance of the food avoidance model classification in patients with IBD and the fitting index of each model, model 3 was finally selected as the optimal model. In the discriminant analysis, the posterior probabilities for each category had mean values between 94.9% and 96.7%, which are all above 90% in Table 2. This showed that the results from the best model found in the LPA were accurate and had much discriminant power.

**TABLE 1:** Latent class model fit comparison.

Model	AIC^a^	BIC^b^	aBIC^c^	Entropy	*p* value		Class probability (%)
					LMR^d^	BLRT^e^	
1	22648.685	22785.013	22670.823	—	—	—	—
2	21692.685	21900.964	21726.508	0.880	< 0.001^∗∗^	< 0.001^∗∗^	0.433/0.567
3	21369.276	21649.506	21414.783	0.889	0.0424^∗^	0.0436^∗^	0.223/0.505/0.272
4	21190.181	21542.362	21247.372	0.882	0.4371	0.4412	0.387/0.181/0.255/0.178
5	21018.919	21443.051	21087.794	0.899	0.2332	0.2361	0.172/0.110/0.233/0.313/0.172

^a^AIC, Akaike information criterion.

^b^BIC, Bayesian information criterion.

^c^aBIC, Adjusted Bayesian information criterion.

^d^LMR, Lo–Mendell–Rubin.

^e^BLRT, Bootstrapped likelihood ratio test.

^∗^ *p* < 0.05.

^∗∗^ *p* < 0.001.

**TABLE 2:** Average attribution probabilities for the three potential categories.

Classification of latent profiles	Probability of belonging to profiles (percentage, %)		
	Class 1	Class 2	Class 3
Class 1	96.7	3.3	0.0
Class 2	2.6	94.9	2.5
Class 3	0.0	4.7	95.3

*Note:* Class 1, mild‐food avoidance adaptation group; Class 2, moderate‐food pleasure deficiency group; Class 3, severe‐food avoidance impairment group.

**FIGURE 3:**
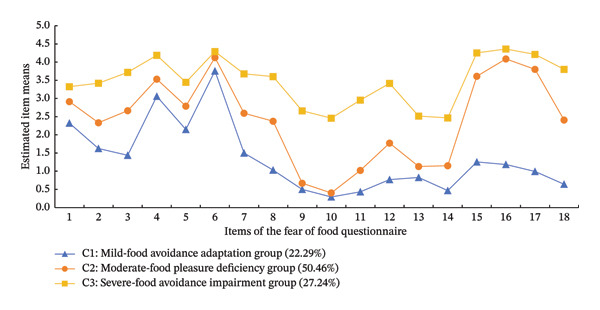
Characteristics of three potential categories of the fear of food questionnaire among patients with inflammatory bowel disease.

Figure 3 displays the average scores for each category in the Chinese version of the FFQ entries. The three categories were named based on the fluctuations in each entry’s average scores. The FFQ score for Category 1 was 23.96 ± 9.45, typically reflective of mild food avoidance. The scores for this category ranged from 0.5 to 3.8, corresponding to responses from “not at all” to “moderately.” This indicates that patients in this category have somewhat adapted to the adverse effects of disease stimuli on dietary management. Consequently, this group was termed the “mild‐food avoidance adaptation group,” comprising 22.29% of the sample. Category 2 recorded an FFQ score of 43.27 ± 9.21, indicating moderate food avoidance. Scores in the pleasure deficit dimension were notably higher, leading to the designation “moderate‐food pleasure deficiency group,” which represented 50.46% of the sample. Category 3 had an FFQ score of 62.84 ± 8.21, surpassing the threshold for severe food avoidance. The high scores on every item led to this subgroup being named the “severe‐food avoidance impairment group,” representing 27.24% of the study sample.

### 3.4. Patient Characteristics

The demographic and disease‐related characteristics of the participants are presented in Table 3.

**TABLE 3:** Classification of latent profiles of general demographic data.

Variables		Total sample (*n* = 323)	Mild‐food avoidance adaptation group (*n* = 72)	Moderate‐food pleasure deficiency group (*n* = 163)	Severe‐food avoidance impairment group (*n* = 88)	*F*/*χ* ^2^	*p* value
Gender	Male	222 (68.7)	51 (70.8)	110 (67.5)	61 (69.3)	0.280	0.844
	Female	101 (31.3)	21 (29.2)	53 (32.5)	27 (30.7)		

Age (year)	18–44	225 (69.7)	51 (70.8)	116 (71.2)	58 (65.9)	1.713	0.788
	45–59	74 (22.9)	17 (23.6)	36 (22.1)	21 (23.9)		
	60–89	24 (7.4)	4 (5.6)	11 (6.7)	9 (10.2)		

Education level	Primary or lower secondary school	85 (26.3)	13 (18.1)	44 (27.0)	28 (31.8)	6.511	0.164
	High school or specialized vocational school	47 (14.6)	8 (11.1)	24 (14.7)	15 (17.0)		
	Associate degree or above	191 (59.1)	51 (70.8)	95 (58.3)	45 (51.1)^a^		

Marital status	Unmarried or divorced	116 (35.9)	26 (36.1)	64 (39.3)	26 (29.5)	2.347	0.309
	Married	207 (64.1)	46 (63.9)	99 (60.7)	62 (70.5)		

Living in an urban area	Yes	281 (87.0)	62 (86.1)	146 (89.6)	73 (83.0)	2.276	0.321
	No	42 (13.0)	10 (13.9)	17 (10.4)	15 (17.0)		

Living alone	Yes	26 (8.0)	6 (8.3)	11 (6.7)	9 (10.2)	0.944	0.624
	No	297 (92.0)	66 (91.7)	152 (93.3)	79 (89.8)		

Employment status	Employment	182 (56.3)	40 (55.6)	92 (56.4)	50 (56.8)	4.436	0.350
	Unemployment	89 (27.6)	17 (23.6)	43 (26.4)	29 (33.0)		
	Student	52 (16.1)	15 (20.8)	28 (17.2)	9 (10.2)		

Monthly household income (yuan)	< 3000	23 (7.1)	6 (8.3)	9 (5.5)	8 (9.1)	7.178	0.305
	3000–5000	78 (24.1)	11 (15.3)	42 (25.8)	25 (28.4)		
	5000–8000	60 (18.6)	12 (16.7)	30 (18.4)	18 (20.5)		
	> 8000	162 (50.2)	43 (59.7)	82 (50.3)	37 (42.0)		

Self‐perceived financial stress	No stress	89 (27.6)	29 (40.3)	45 (27.6)	15 (17.0)^a^	12.208	0.016^∗^
	Moderate stress	155 (48.0)	32 (44.4)	77 (47.2)	46 (52.3)		
	High stress	79 (24.5)	11 (15.3)	41 (25.2)	27 (30.7)		

Type of disease	CD	259 (80.2)	57 (79.2)	136 (83.4)	66 (75.0)	2.620	0.270
	UC	64 (19.8)	15 (20.8)	27 (16.6)	22 (25.0)		

Current disease status	Remission stage	216 (66.9)	59 (81.9)	119 (73.0)	38 (43.2)^ab^	32.446	< 0.001^∗∗^
	Active stage	107 (33.1)	13 (18.1)	44 (27.0)	50 (56.8)^ab^		

Years of diagnosis (year)	< 1	89 (27.6)	13 (18.1)	54 (33.1)	22 (25.0)	6.878	0.142
	1–5	150 (46.4)	36 (50.0)	69 (42.3)	45 (51.1)		
	> 5	84 (26.0)	23 (31.9)	40 (24.5)	21 (23.9)		

Bowel surgery status	Yes	274 (84.8)	64 (88.9)	134 (82.2)	76 (86.4)	1.953	0.377
	No	49 (15.2)	8 (11.1)	29 (17.8)	12 (13.6)		

Use of biologic therapy	Yes	185 (57.3)	49 (68.1)	99 (60.7)	37 (42.0)^ab^	12.558	0.002^∗^
	No	138 (42.7)	23 (31.9)	64 (39.3)	51 (58.0)^ab^		

Nutritional support therapy	Yes	141 (43.7)	25 (34.7)	72 (44.2)	44 (50.0)	3.794	0.150
	No	182 (56.3)	47 (65.3)	91 (55.8)	44 (50.0)		

Malnutrition risk	Yes	117 (36.2)	21 (29.2)	55 (33.7)	41 (46.6)	6.081	0.048^∗^
	No	206 (63.8)	51 (70.8)	108 (66.3)	47 (53.4)		

Food tolerance tests	Yes	50 (15.5)	7 (9.7)	24 (14.7)	19 (21.6)	4.407	0.110
	No	273 (84.5)	65 (90.3)	139 (85.3)	69 (78.4)		

^a^ *p* < 0.05 compared to the mild‐food avoidance adaptation group.

^b^ *p* < 0.05 compared to the moderate‐food pleasure deficiency group.

^∗^ *p* < 0.05.

^∗∗^ *p* < 0.001.

### 3.5. Comparison of Food Avoidance Among Latent Classes

A total of 302 valid questionnaires were analyzed for the food avoidance category among patients with IBD, as described previously.

The “mild‐food avoidance adaptation group” avoided an average of 2.78 ± 2.37 food categories; the “moderate‐food pleasure deficiency group” avoided an average of 3.59 ± 2.49 food categories; and the “severe‐food avoidance impairment group” avoided an average of 3.89 ± 2.51 food categories, with a statistical significance of *p* = 0.02, as shown in Table 4.

**TABLE 4:** Food choice of the latent profiles of food avoidance.

Food avoidance categories	Avoidance frequency (*n* = 302)	Mild‐food avoidance adaptation group (*n* = 67)	Moderate‐food pleasure deficiency group (*n* = 156)	Severe‐food avoidance impairment group (*n* = 79)
Seafood	224 (74.2)	40 (59.7)	123 (78.8)	61 (77.2)
Dairy products	161 (53.3)	29 (43.3)	82 (52.6)	50 (63.3)
Nut foods	158 (52.3)	25 (37.3)	86 (55.1)	47 (59.5)
Coarse grains	144 (47.7)	28 (41.8)	71 (45.5)	45 (57.0)
Edible Fungi	90 (29.9)	10 (14.9)	53 (34.0)	27 (34.6)
Porridge or rice soup	41 (13.6)	12 (17.9)	24 (15.4)	5 (6.3)
Root vegetables	38 (12.6)	5 (7.5)	20 (12.8)	13 (16.5)
Fruits	34 (11.3)	5 (7.5)	19 (12.2)	10 (12.7)
Bean products	34 (11.3)	5 (7.5)	16 (10.3)	13 (16.5)
Freshwater aquatic animals	27 (8.9)	8 (11.9)	10 (6.4)	9 (11.4)
Rice	23 (7.6)	1 (1.5)	13 (8.3)	9 (11.4)
Stuffed foods	23 (7.6)	4 (6.0)	13 (8.3)	6 (7.6)
Dark‐colored vegetables	20 (6.6)	2 (3.0)	8 (5.1)	10 (12.7)
Light‐colored vegetables	18 (6.0)	4 (6.0)	10 (6.4)	4 (5.1)
Poultry	15 (5.0)	2 (3.0)	5 (3.2)	8 (10.1)
Flour‐based foods	9 (3.0)	2 (3.0)	6 (3.8)	1 (1.3)
Eggs	8 (2.6)	1 (1.5)	4 (2.6)	3 (3.8)
Red meat	8 (2.6)	2 (3.0)	4 (2.6)	2 (2.5)

### 3.6. Univariate Analysis of Food Avoidance

Table 3 shows significant differences (*p* < 0.05) in self‐perceived financial stress (*χ* ^2^ = 12.208, *p* = 0.016), current disease status (*χ* ^2^ = 32.446, *p* < 0.001), use of biologic therapy (*χ* ^2^ = 12.558, *p* = 0.002) and malnutrition risk (*χ* ^2^ = 6.081, *p* = 0.048) among the three patient categories of the univariate analysis. Table 5 shows significant differences in scores of FoP‐Q‐SF (*F* = 57.113, *p* < 0.001), IPQ‐B (*F* = 36.027, *p* < 0.001), and IBD‐SES (*F* = 15.692, *p* < 0.001) between the three patient categories.

**TABLE 5:** Differences in symptom scores across different latent profiles.

Variables	Total points	Classification of latent profiles			Multiple comparisons	*F*	*p*
		Mild‐food avoidance adaptation group (*n* = 72)	Moderate‐food pleasure deficiency group (*n* = 163)	Severe‐food avoidance impairment group (*n* = 88)			
Fear of Progression Questionnaire Short Form	31.12 ± 10.45	23.92 ± 7.45	30.10 ± 8.72^a^	38.91 ± 10.52^ab^	Class 1 < Class 2 < Class 3	57.113	< 0.001^∗∗^
Brief Illness Perception Questionnaire	42.84 ± 7.66	37.19 ± 7.16	43.41 ± 7.09^a^	46.41 ± 6.46^ab^	Class 1 < Class 2 < Class 3	36.027	< 0.001^∗∗^
Inflammatory Bowel Disease‐Self‐Efficacy Scale	186.55 ± 31.91	198.57 ± 29.05	188.96 ± 29.84^a^	172.27 ± 32.94^ab^	Class 1 > Class 2 > Class 3	15.692	< 0.001^∗∗^
Social Support Rating Scale	38.16 ± 7.80	38.86 ± 7.71	38.61 ± 7.55	36.76 ± 8.22	—	1.994	0.138

*Note:* Class 1, mild‐food avoidance adaptation group; Class 2, moderate‐food pleasure deficiency group; Class 3, severe‐food avoidance impairment group. —Indicates that no such data exists.

^a^ *p* < 0.05 compared to the mild‐food avoidance adaptation group.

^b^ *p* < 0.05 compared to the moderate‐food pleasure deficiency group.

^∗∗^ *p* < 0.001.

### 3.7. Multicollinearity Testing

Seven variables were identified as statistically significant in the single‐factor analysis of variance. They were subjected to the covariance test, and the VIFs were all < 5, suggesting no multicollinearity between the variables.

### 3.8. Multinomial Logistic Regression Analysis of Food Avoidance

With the category of the potential profile of food avoidance in patients with IBD as the dependent variable, the “mild‐food avoidance adaptation group,” “moderate‐food pleasure deficiency group,” and “severe‐food avoidance impairment group” were assigned the values of 1, 2, and 3, respectively. The statistically significant items in the univariate analysis were used as independent variables for multiple logistic regression analyses. The methods of independent variable assignment were the Chinese version of FoP‐Q‐SF, IPQ‐B, SSRS, and IBD‐SES total score were carried in actual values; self‐perceived financial stress, no stress (*Z*_1_ = 1, *Z*_2_ = 0), moderate stress (*Z*_1_ = 0, *Z*_2_ = 1), and high stress (*Z*_1_ = 0, *Z*_2_ = 0); current disease status, active stage = 0, remission stage = 1; use of biologic therapy, yes = 1, no = 2; and malnutrition risk, yes = 1, no = 2.

The influencing factors of food avoidance on different groups of patients with IBD are shown in Table 6. Multinomial logistic regression analysis revealed that, compared with Class 1 (mild‐food avoidance adaptation group), patients with higher levels of fear of disease progression (FoP‐Q‐SF) and more negative illness perceptions (IPQ‐B) were more likely to be classified into Class 2 (moderate‐food pleasure deficiency group) (FoP‐Q‐SF: OR = 1.070, 95% CI: 1.022–1.119, *p* = 0.004; IPQ‐B: OR = 1.108, 95% CI: 1.053–1.165, *p* < 0.001) and Class 3 (severe‐food avoidance impairment group) (FoP‐Q‐SF: OR = 1.169, 95% CI: 1.108–1.234, *p* < 0.001; IPQ‐B: OR = 1.106, 95% CI: 1.037–1.180, *p* = 0.002). In addition, compared with Class 3, patients in Class 2 reported significantly lower levels of fear of disease progression (FoP‐Q‐SF: OR = 0.915, 95% CI: 0.882–0.949, *p* < 0.001). Regarding disease status, patients in remission were more likely to belong to Class 1 (OR = 0.328, 95% CI: 0.121–0.893, *p* = 0.029) and Class 2 (OR = 3.132, 95% CI: 1.535–6.387, *p* = 0.002), compared to those in Class 3.

**TABLE 6:** Multinomial logistic regression analysis of latent profiles of food avoidance.

Variables	*β*	Standard error	Warld *x* ^2^	*p*	OR	OR [95% CI]
Class 2 (ref.: Class 1)						
Fear of Progression Questionnaire Short Form	0.067	0.023	8.406	0.004	1.070	1.022∼1.119
Brief Illness Perception Questionnaire	0.102	0.026	15.642	< 0.001	1.108	1.053∼1.165
Class 3 (ref.: Class 1)						
Current disease status (ref.: Active stage)						
Remission stage	−1.114	0.51	4.765	0.029	0.328	0.121∼0.893
Fear of Progression Questionnaire Short Form	0.156	0.028	31.95	< 0.001	1.169	1.108∼1.234
Brief Illness Perception Questionnaire	0.101	0.033	9.433	0.002	1.106	1.037∼1.180
Class 2 (ref.: Class 3)						
Current disease status (ref.: Active stage)						
Remission stage	1.142	0.364	9.855	0.002	3.132	1.535∼6.387
Fear of Progression Questionnaire Short Form	−0.089	0.019	22.786	< 0.001	0.915	0.882∼0.949

*Note:* Class 1 for the mild‐food avoidance adaptation group, Class 2 for the moderate‐food pleasure deficiency group, Class 3 for the severe‐food avoidance impairment group.

## 4. Discussion

### 4.1. The Subgroups of Food Avoidance Among Patients With IBD

The study indicated that the average of FFQ among patients with IBD was 44.22 ± 16.38, corresponding to the “moderate” qualitative food avoidance severity range. Additionally, LPA revealed three distinct profiles of food avoidance among patients with IBD, underscoring significant heterogeneity within this population.

First, the mild‐food avoidance adaptation group (22.29%) demonstrated the lowest FFQ scores (23.96 ± 9.45), suggesting a relatively adaptive approach to dietary management with minimal restriction. These patients may have effectively integrated disease‐related dietary adjustments without excessive avoidance. Then, the moderate‐food pleasure deficiency group (50.46%) had moderate FFQ scores (43.27 ± 9.21) and emerged as the most prevalent profile, indicating that food‐related fear, avoidance, and pleasure loss are common experiences. Finally, the severe‐food avoidance impairment group (27.24%) displayed high FFQ scores (62.84 ± 8.21), reflecting substantial psychosocial impairment and significant avoidance behaviors. Identifying these distinct avoidance profiles highlights the need for individualized clinical interventions. Healthcare providers should especially focus on patients in the moderate and severe avoidance groups, addressing not only immediate nutritional requirements and symptom management but also the ongoing psychosocial consequences. Such psychological implications of food avoidance may persist despite symptom remission, potentially resulting in prolonged nutritional deficiencies and decreased quality of life. Furthermore, nursing managers should encourage interdisciplinary collaboration involving dietitians and mental health professionals to develop personalized nutritional and psychological support plans. Educational programs aimed at managing food‐related anxiety and providing culturally tailored dietary guidance may further reduce unnecessary avoidance behaviors, ultimately enhancing patient outcomes.

### 4.2. The Pattern of Food Avoidance Among Patients With IBD

This study found that, after excluding food allergies, food intolerances, and objectively harmful foods, patients with IBD still avoided an average of 3.49 ± 2.49 food categories over the past 3 months. Although lower than previously reported findings [5, 45], notable avoidance remained, particularly toward seafood (74.2%), dairy products (53.3%), nut foods (52.3%), and coarse grains (47.7%). Differing from dietary avoidance patterns in Western IBD populations, Chinese patients exhibited notably higher avoidance of seafood and significantly lower avoidance of red meats. The reasons for this divergence have been clarified through related qualitative research conducted by our team [46]. Specifically, Chinese patients commonly categorize seafood as a “trigger food” causing disease relapse, a belief intensified by concerns regarding seafood freshness after long‐distance transportation across Chinese vast territory. Conversely, limited awareness about the proinflammatory nature of pork, coupled with pork’s longstanding cultural significance, affordability, and availability in China, results in its infrequent avoidance. Further analyses demonstrated that the three latent classes of food avoidance among patients with IBD exhibited a progressively broader range from mild to severe impairment. Compared with the mild‐food avoidance adaptation group, the moderate‐food pleasure deficiency and severe‐food avoidance impairment groups showed significantly more extensive dietary restrictions. Notably, patients in the severe group had avoidance rates exceeding 10% across 12 food categories, including rice and poultry. Consequently, nurses should adopt individualized nutritional management strategies in clinical practice. Specifically, comprehensive dietary assessments and structured educational interventions should be implemented to correct misconceptions regarding food safety, freshness, and inflammatory potential. These strategies aim to reduce unnecessary dietary avoidance behaviors, enhance nutritional intake, and ultimately improve patient prognosis and quality of life.

### 4.3. Factors Influencing Food Avoidance Among Patients With IBD

This study identified the phase of IBD activity as a key determinant in patients’ classification into different latent food avoidance groups. Consistent with previous findings, patients in the active phase were more likely to belong to high‐avoidance food classes (3.01 ± 2.39 vs. 4.46 ± 2.42), while those in remission exhibited fewer dietary restrictions, but avoidance behaviors remained common nonetheless [16]. This pattern diverges from clinical guidelines that advocate for adequate and balanced nutrition [30]. During flare‐ups, patients often attribute worsening gastrointestinal symptoms to recently consumed foods and respond by avoiding these perceived “trigger foods” [47]. Although such reactive dietary adjustments may be understandable, they are frequently made without professional guidance. Over time, with recurrent symptoms, patients may expand their list of restricted foods unnecessarily, leading to excessive food avoidance and an increased risk of malnutrition [7]. These findings suggest that the active disease phase represents a critical period during which patients’ dietary patterns are most restricted and require clinical attention. Nurses should remain vigilant regarding restrictive eating behaviors in patients during active IBD episodes, routinely assess their dietary habits and nutritional status, and establish early referral mechanisms to dietitians. Timely professional dietary guidance can help patients relieve symptoms while ensuring adequate nutritional intake, preventing complications associated with unnecessary dietary restriction.

Compared to the other two groups, patients with higher levels of fear of disease progression were more likely to be classified as the severe‐food avoidance impairment group. This finding supports previous research showing that approximately 77% of patients with IBD report avoiding certain foods out of fear of disease relapse or worsening [7]. Such fears are often reinforced by past experiences of symptom exacerbation following specific food intake, leading to progressively more restrictive and overly cautious eating behaviors [12]. In the absence of a structured food reintroduction plan, patients may become trapped in a limited and nutritionally inadequate dietary pattern, which may negatively affect both physical and psychological well‐being [48]. These results highlight that managing patients’ anxiety regarding disease progression is a critical step in preventing maladaptive food avoidance. In clinical practice, healthcare professionals should implement structured educational interventions to help patients develop accurate dietary beliefs and replace irrational food fears with evidence‐based knowledge. Nurse managers should facilitate the establishment of multidisciplinary care pathways that involve both dietitians and mental health professionals. Dietitians can guide patients through a controlled and gradual reintroduction of previously avoided foods, while psychologists can apply cognitive‐behavioral therapy to help patients identify and manage food‐related anxiety. Such integrated nursing interventions can support patients in rebuilding a more balanced and nutritionally sufficient diet, improving overall nutritional status, reducing avoidance behaviors driven by fear, and enhancing quality of life.

This study found that higher levels of negative disease perception predicted a greater likelihood of falling into the moderate‐food pleasure deficiency group and the severe‐food avoidance impairment group. In other words, how patients perceive and emotionally respond to their illness plays a key role in shaping their dietary management strategies. Patients who hold a pessimistic view of their condition are more likely to adopt food avoidance as a means of coping with what they perceive to be an uncontrollable disease [48]. This pattern may stem from an overestimation of the link between food intake and symptom exacerbation [12], as well as limited access to timely, professional dietary counseling in clinical practice [49]. These findings offer important implications for clinical nursing: the cognitive representation of patients with IBD can be just as influential as their physical symptoms in determining disease self‐management behaviors. Nurses should routinely assess patients’ illness perceptions using validated tools as part of individualized dietary counseling. When negative or distorted beliefs about disease and diet are identified, nurses should provide targeted health education to address these misconceptions, for example, by clarifying the actual role of specific foods in symptom management. Nurse managers should organize regular training for staff on empathetic communication and cognitive reframing techniques to enhance patient‐centered care. In cases of persistent food‐related anxiety or depressive symptoms, nurse leaders should establish clear referral pathways to dietitians and mental health professionals for cognitive‐behavioral interventions. Integrating these strategies into routine nursing practice can help reduce fear‐driven food avoidance and promote more balanced dietary patterns among patients with IBD.

## 5. Conclusion

Food avoidance is prevalent among patients with IBD, with significant variation across individuals. This study identified three distinct latent profiles, including the mild‐food avoidance adaptation group, the moderate‐food pleasure deficiency group, and the severe‐food avoidance impairment group, with the mild‐food avoidance adaptation group being the most common, with the members avoiding an average of three food categories. Food avoidance was influenced by fear of disease progression, negative illness perceptions, and disease remission status. Patients with greater fear or negative cognitive appraisal of their illness were more likely to exhibit severe food avoidance, whereas those in remission tended to show milder patterns. To improve dietary management, healthcare professionals should deliver phase‐specific and personalized nutritional counseling, integrate psychological support, and provide culturally sensitive education to correct misconceptions and reduce unnecessary food restrictions, thereby promoting better nutritional outcomes and quality of life.

## 6. Limitations

This study has several limitations. First, all participants were recruited from a single province in eastern China. Regional variation in dietary culture, healthcare access, and educational background may limit the generalizability of the findings. Future studies should include more diverse geographical samples to enhance representativeness. Second, this study focused solely on hospitalized patients with IBD, who may experience more severe symptoms and higher levels of food avoidance compared to community‐dwelling patients. Further research should incorporate outpatient or population‐based samples to capture a broader spectrum of disease experiences. Third, the cross‐sectional design prevents causal inferences between disease activity, illness perception, and food avoidance behaviors. Longitudinal studies are needed to explore how food avoidance patterns evolve over time. Finally, children and adolescents with IBD were not included. Given their heightened vulnerability to nutritional deficiency and psychosocial challenges, future research should examine food avoidance behaviors across developmental stages.

## 7. Implications for Nursing Management

This study, grounded in the Roy adaptation model and employing LPA, identified three distinct food avoidance subtypes and their characteristics among patients with IBD. To address the complexity of dietary behaviors in this population, nursing management should strengthen efforts in patient assessment, dietary intervention, and referral pathways. First, in terms of assessment, brief and validated screening tools should be incorporated into routine clinical evaluations to identify food avoidance behaviors early, particularly during active disease phases. Second, regarding intervention, nursing managers should develop multidisciplinary care pathways that involve dietitians and mental health professionals. Nurses should be trained to conduct individualized nutritional assessments and deliver structured educational interventions to correct misconceptions about diet and disease. Finally, for referral systems, clear protocols should be established to ensure that patients experiencing persistent food‐related anxiety or emotional distress are referred for cognitive‐behavioral interventions. These strategies can enhance patients’ adaptive capacity, improve nutritional outcomes, and promote overall quality of life.

## Author Contributions

Study concept and design: Qingyu Wang, Hana F. Zickgraf, Zheng Lin, and Yang Lei. Acquisition of data: Qingyu Wang and Jiefeng Yang. Statistical analysis: Qingyu Wang and Sha Li. Interpretation of data: Qingyu Wang and Meijing Zhou. Drafting the manuscript: Qingyu Wang. Critical revision of the manuscript for important intellectual content: Hana F. Zickgraf, Zheng Lin, and Yang Lei. Obtained funding: Zheng Lin and Yang Lei. Study supervision: Hana F. Zickgraf, Zheng Lin, and Yang Lei.

## Funding

This work was supported by the “333 High‐Level Talents Training Project” of Jiangsu Province (grant number BRA2020069), National Natural Science Foundation of China (grant number 82204167), and the Qingfeng Scientific Research Fund of the China Crohns & Colitis Foundation CCCF (grant number CCCF‐QF‐2023C49‐4).

## Disclosure

All authors read and approved the final manuscript.

## Ethics Statement

It was approved by the Institutional Ethics Committee of Nanjing Medical University (No.2022955).

## Conflicts of Interest

The authors declare no conflicts of interest.

## Data Availability

The data that support the findings of this study are available from the corresponding authors upon reasonable request.
